# Expression and function of transmembrane 4 superfamily proteins in digestive system cancers

**DOI:** 10.1186/s12935-020-01353-1

**Published:** 2020-07-16

**Authors:** Yaoyue Qi, Hui Li, Jing Lv, Weiwei Qi, Liwei Shen, Shihai Liu, Aiping Ding, Gongjun Wang, Libin Sun, Wensheng Qiu

**Affiliations:** 1 Qingdao University, Qingdao, Shandong China; 2 Department of Oncology, Affiliated Hospital of Qingdao University, Qingdao, Shandong China; 3 Department of Oncology, Qingdao Women and Children’s Hospital, Qingdao, Shandong China; 4 Central Laboratory, Affiliated Hospital of Qingdao University, Qingdao, Shandong China

**Keywords:** Transmembrane 4 superfamily, Digestive system cancers, Bioinformatics analysis, Proliferation, Metastasis

## Abstract

**Background:**

Although the medical level is constantly improving, cancer is still a major disease that threatens human health, and very effective treatments have not been found. In recent years, studies have found that four-transmembrane superfamily proteins are involved in multiple stages of tumorigenesis and development, but their expression and function in tumors have not been systematically studied.

**Methods:**

We used the Oncomine database to analyze the mRNA expression levels of TSPAN family in various cancers. Then differentially expressed genes were screened out and verified by liver cancer, colorectal cancer, and gastric cancer cells by q-PCR and Western blot analysis. CCK8 and EDU analysis are used to detect cell proliferation, Cell wound scrape assay and Cell invasion assay are used to analyze cell invasion and metastasis. Nude tumor formation test used to verify the tumor suppressive effect of TSPAN7 in vivo.

**Results:**

Differential analysis of 33 TSPAN proteins revealed that a total of 11 proteins showed differential expression in 10% of independent analyses, namely TSPAN1, TSPAN3, TSPAN5, TSPAN6, TSPAN7, TSPAN8, TSPAN13, TSPAN25, TSPAN26, TSPAN29, TSPAN30. TSPAN7 is the only four-transmembrane protein with reduced expression in three types of digestive tract tumors, so we chose TSPAN7 to be selected for cellular and molecular level verification. We found that compared with normal cells, the expression of TSPAN7 in liver cancer cells was significantly reduced, while the expression of gastric and colon cancer was not significantly different from that of normal cells. In addition, we also found that the high expression of Tspan7 not only inhibited the proliferation of HCC-LM3 cells, but also inhibited its invasion and metastasis.

**Conclusions:**

Our study evaluated the expression and function of the TSPANs family in digestive cancers and explored TSPAN7 in hepatoma cells in detail. We found some members of the TSPAN family show significant expression differences between cancer and normal tissues, of which TSPAN7 may be a potential biomarker for liver cancer.

## Background

Tetraspanins (TSPANs) are a family of proteins with four transmembrane domains that are found in all multicellular eukaryotes [1]. Tetraspanins are highly conserved among species, and 34 tetraspan membrane proteins have been found in mammals, of which 33 have been identified in humans [2]. The transmembrane domains of the TSPAN family are as follows: a small extracellular domain (SEL), a large extracellular loop (LEL), and the intracellular N- and C-termini [3]. Four-transmembrane proteins are generally considered scaffold proteins, which can anchor multiple proteins to a region of the cell membrane to form four-transmembrane protein-enriched microdomains (TEMs), thereby transducing signals [4, 5]. TSPANs are a group of evolutionarily conserved transmembrane proteins, but there are small variable domains in their LELs, which may be one of the reasons for the functional differences between isotypes [2]. Increasing evidence shows that four-transmembrane proteins can regulate a variety of biological processes, including cell migration, adhesion, activation, and apoptosis [6–8]. In addition, some members of the four-transmembrane protein family, such as TSPAN [1] and TSPAN13, have been shown to affect tumor metastasis and progression, but there are few studies on these functions [9, 10]. Therefore, this study will analyze the differences in TSPAN family expression in cancer patients and normal populations through a public database and will evaluate their prognostic value.

Cancer has been a popular research area in recent years due to its increasing morbidity and mortality [11]. In China, the five most common types of cancer are lung cancer, gastric cancer, liver cancer, colorectal cancer, and esophageal cancer, of which digestive tract tumors account for a large proportion [12]. This study aimed to evaluate the expression and function of the TSPANs family in digestive cancers. After summarizing the TSPAN family and its different correlations in various types of tumorigenesis, this study explored TSPAN7 in hepatoma cells in detail and concluded that TSPAN7 may be a potential biomarker for liver cancer.

## Materials and methods

### Oncomine database analysis

The online public database Oncomine (http://www.oncomine.org) was used to identify the mRNA expression of TSPAN family members in both tumor and normal tissues in common human tumors [13]. For each cancer and gene, the thresholds were set as follows: p-value: 0.01; fold change: 2; gene rank: 10%; analysis type: cancer vs. normal; data type: mRNA.

### Cells and culture conditions

The normal gastric epithelial cell line GES-1, the gastric cancer cell lines AGS and HGC27, the normal liver cell line 7702, the liver cancer cell lines HCC-LM3 and HepG2, the normal colon epithelial cell line NCM460, and the colon cancer cell lines SW480 and SW620 were purchased from the Shanghai Institute of Biochemistry and Cell Biology (Shanghai, China). The cell lines were all cultured in medium supplemented with 10% fetal bovine serum (FBS) at 37 °C with 95% humidity and 5% CO_2_. Cell culture was performed as described previously [14].

### Transfection with lentiviral particles

The cells were seeded at a density of 2 × 105 cells/well in six-well plates, and 2 ml of complete medium was added to each well. The cells were incubated for 24 h and infected with lentiviral particles, and 12 h after infection, the LV-containing medium was replaced with fresh complete medium. The infected cells were then selected with 4 μg/ml puromycin for 96 h. Empty lentiviral vector was used as a control [15]. The lentiviral expression vectors LV-ctrl, LV-TSPAN7, LV-shTSPAN7 were purchased from Shanghai Gene Pharma Company (China).Virus packaging involves three plasmids, which are the tool vector plasmid (GV115, GV118, GV365) carrying the target gene or target sequence, the virus packaging auxiliary plasmid Helper 1.0 and the virus packaging auxiliary plasmid Helper 2.0 (Additional file 1).

### Real-time PCR

Total RNA was extracted from the cells by TRIzol (Takara). Primer Buffer (5×), Primase, Oligonucleotide DT Primer and Reverse Transcriptase (TAKARA) were used to convert 1 µg of RNA into DNA. PCR conditions were as follows: 10 min at 95 °C; 45 cycles of 10 s at 95 °C, 20 s at 60 °C and 10 s at 72 °C. Real-time PCR was performed using a SYBR GREEN PCR mixture, and gene expression was standardized for GAPDH. Each experiment was repeated three times independently. The cyclic threshold method (CT) was used to quantify mRNA expression. SPSS11.5 software was used to calculate the significant difference in mRNA expression levels between different samples. The relative amount of target genes was carried out using the 2− ΔΔCt method while GAPDH was used as internal control. Real-time PCR was performed as described previously [16]. The primer sequences for qRT-PCR were as follows: TSPAN7 forward, ACACGGACGCTATGCAGAC, and reverse, CCTGGGGATTACAATCAGTTTCG; GAPDH forward, GGAGCGAGATCCCTCCAAAAT, and reverse, GGCTGTTGTCATACTTCTCATGG.

### Western blot analysis

Cells were lysed on ice in RIPA buffer containing PMSF, and then the mix was centrifuged at 13,000×*g* for 5 min at 4 °C to remove cell debris. The supernatant was collected, and a BCA protein assay kit was used to determine the total protein concentration. Approximately 20 µg of protein was separated by 15% sodium lauryl sulfate–polyacrylamide gel electrophoresis and then transferred to a polyvinylidene fluoride membrane. The membrane was blocked in 5% skim milk and then incubated with a primary antibody against β-actin-HPR (120,000, Sigma). A Tspan7 antibody (1: 1000, Abcam) was added and incubated at 4 °C overnight. Subsequently, the cells were incubated with secondary antibodies (120,000, Abcam) for 1 h at room temperature. Chemiluminescence was used to observe the antibody staining. Western blot was performed as described previously [17].

### Cell viability assay

After digestion with 0.25% EDTA-trypsin, cells were seeded at a density of 5000/well into 96-well plates, and cell proliferation was measured at 24, 48, and 72 h using Cell Counting Kit-8. Briefly, 10 µl of CCK-8 solution was added to each well and incubated with the cells at 37 °C for 2 h. Optical density (OD) was then measured at 450 nm with a microplate spectrophotometer [18]. The cells were seeded in a six-well plate and were incubated overnight, after which EdU detection reagent was added, and cell proliferation was observed with a fluorescence inverted microscope (Olympus, Japan) [19].

### Scratch assay

Cells were seeded into 6-well plates at 90% density. Then, the cell layer was scratched with the tip of a 200 µl pipette to form a wound along the center of each well. Next, the wells were washed twice with PBS to remove floating cells, and fresh medium was added. Images were captured at 0 and 24 h (100× magnification) to assess cell migration into the wound area [20].

### Cell invasion assay

Transwell cell culture chambers containing Matrigel were used for invasive evaluation. Log phase cells were digested with 0.25% EDTA-trypsin, and the cell suspension was treated with serum-free DMEM. Then, 200 μL of cells was added to the upper cavity of the Transwell chamber, and 600 µl of medium containing 20% serum was added to the lower part of the well. After 24 h of incubation, the cells were fixed with methanol for 30 min and then were stained with Giemsa for 20 min. The cells remaining in the upper cavity were gently removed with a wet cotton swab and then were placed under an inverted microscope so that the remaining cells could be counted [21].

### In vivo xenograft study

Animal studies were carried out in strict adherence with institutional guidelines. HCC-LM3 cells (2 × 106/200 µl per mouse) were subcutaneously injected into the right hind legs of 6–8 week-old female nude mice. When tumors volume reached 50 mm^3^, the mice were randomized to 3 groups and dosing was initiated. They were: (i) control (vehicle only); (ii) LV-TSPAN7 (100 µg/kg intratumoral injection); (iii) LV-shTSPAN7 (100 µg/kg intratumoral injection). All groups were treated once every 3 days for 40 days. The tumor size and weight were monitored three times a week. Tumor volume (V) was calculated as V = 0.5 × length × width [2, 22].

### Statistical methods

All data are expressed as the mean ± SD. Statistical tests were performed using SPSS 17.0 software (SPSS Inc., Chicago, IL, USA). Student’s t tests were used to determine significant differences between two groups. p < 0.05 was considered significant.

## Results

### Expression levels of the TSPAN family members in human cancers

**Fig. 1:**
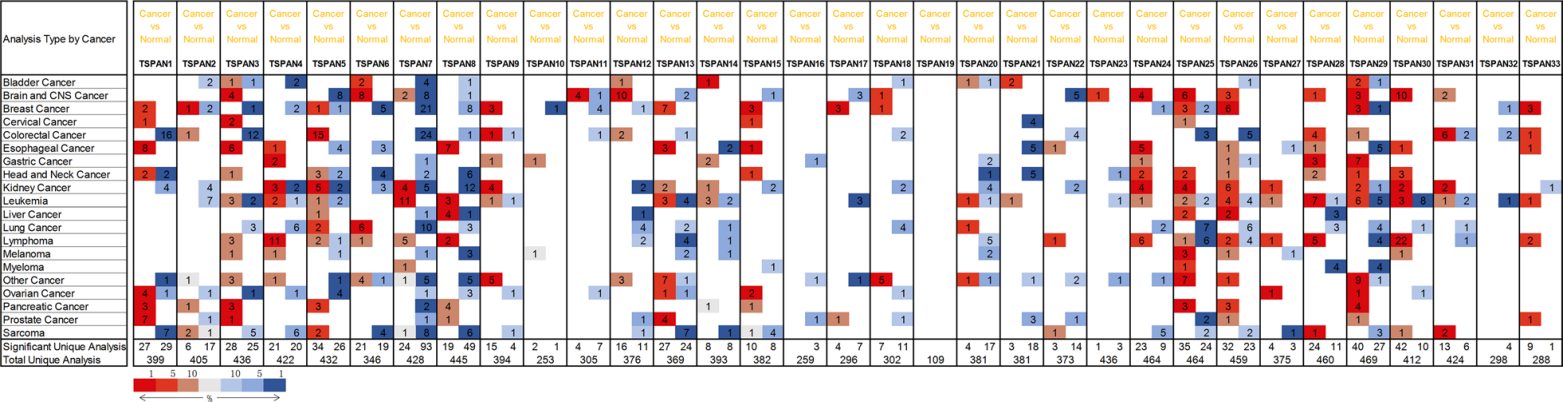
Expression levels of the TSPAN family members in human cancers. The number in the colored cell represents the number of analyses that met the thresholds. The cell color is determined by the gene rank. The more intense colors of red (overexpression) or blue (underexpression) indicate a more highly significantly overexpressed or underexpressed gene, respectively

To investigate whether TSPAN family proteins are differentially expressed between tumor patients and normal populations, we analyzed the expression of 33 members of the four-transmembrane protein family in 21 common tumors through the Oncomine database. Differential analysis of 33 TSPAN proteins revealed that a total of 11 proteins showed differential expression in 10% of independent analyses, namely, TSPAN1, TSPAN3, TSPAN5, TSPAN6, TSPAN7, TSPAN8, TSPAN13, TSPAN25, TSPAN26, TSPAN29, and TSPAN30 (Fig. 1). According to the 2018 Global Cancer Statistics Report, there were approximately 18.1 million new cancer cases and 9.6 million cancer deaths in 2018 [13]. Among them, gastrointestinal tumors account for a large proportion. Combining the results of bioinformatics analysis, we focused on TSPAN protein expression and prognosis in three common digestive tract tumors: liver cancer, intestinal cancer and gastric cancer.

### Expression of the TSPAN family members in liver cancer

The differentially expressed genes in hepatocellular carcinoma were analyzed in the Oncomine database. Among four data sets, it was found that the expression of TSPAN7, TSPAN12, and TSPAN28 was lower in hepatocellular carcinoma patients than it was in the normal population. According to Wurmbach’s study, we learned that TSPAN7 and TSPAN12 are reduced in hepatocellular carcinoma. Through research by Chen and Roessler, we found that TSPAN28 was expressed at low levels in hepatocellular carcinoma, but no other genes were found to have statistically significant differential expression (Table 1).

**Table 1:** Significant changes of TSPANs expression in transcription level between HCC and normal liver tissues (ONCOMINE)

Gene	Dataset	Normal (cases)	Tumor (cases)	Fold change	T-test	p-value
TSPAN7	Wurmbach	Liver (10)	Hepatocellular carcinoma (35)	− 3.244	− 5.760	5.16E–7
TSPAN12	Wurmbach	Liver (10)	Hepatocellular carcinoma (35)	− 3.140	− 7.514	1.48E–9
TSPAN28	Chen	Liver (76)	Hepatocellular carcinoma (104)	− 2.654	− 11.415	1.02E–22
	Roessler 2	Liver (220)	Hepatocellular carcinoma (225)	− 2.279	− 14.950	4.14E–38
	Roessler	Liver (21)	Hepatocellular carcinoma (22)	− 2.615	− 5.165	1.36E–5

HCC: hepatocellular carcinoma

### Expression of the TSPAN family members in colorectal cancer

We analyzed the expression of TSPANs in colorectal cancer through the Oncomine database. As far as TSPAN1 is concerned, 8 data points in 14 sets of data that showed differential expression between colorectal cancer patients and the normal population. Additionally, the genes that were highly expressed in colorectal patients are TSPAN2, TSPAN5, TSPAN12, TSPAN28, TSPAN29, and TSPAN33. Conversely, the low-expression genes were TSPAN1, TSPAN3, TSPAN7, TSPAN8, TSPAN11, TSPAN13, TSPAN18, TSPAN22, TSPAN25, TSPAN26 and TSPAN32. Interestingly, the analysis of the TCGA database found that the expression of TSPAN9 in colorectal cancer patients was lower than it was in the normal population and that the expression of TSPAN31 was higher than it was in the normal population; however, studies by Ki and Skrzypczak2 and others have reached the opposite conclusion (Table 2).

**Table 2:** Significant changes of TSPANs expression in transcription level between CRC and normal colorectal tissues (ONCOMINE)

Gene	Dataset	Normal (cases)	Tumor (cases)	Fold change	T-test	p-value
TSPAN1	Hong	Colon (12)	Colorectal carcinoma (70)	− 11.253	− 20.031	3.31E–33
	Alon	Colon (22)	Colon adenocarcinoma (39)	− 2.360	− 4.048	1.06E–4
	Graudens	Colon (12)	Colorectal carcinoma (18)	− 3.965	− 7.607	5.19E–8
	Kaiser	Colon (5)	Rectosigmoid adenocarcinoma (10)	− 3.848	− 8.900	5.93E–7
			Colon adenocarcinoma (41)	− 3.030	− 11.170	4.07E–10
			Cecum adenocarcinoma (17)	− 3.448	− 7.671	1.19E–7
			Colon mucinous adenocarcinoma (13)	− 2.315	− 6.096	7.91E–6
			Rectal adenocarcinoma (8)	− 3.967	− 4.779	7.70E–4
	Notterman	Colon (18)	Colon adenocarcinoma (18)	− 5.254	− 4.381	1.56E–4
	Skrzypczak 2	Colon (10)	Colon carcinoma (5)	− 4.128	− 16.832	1.23E–9
			Colon carcinoma epithelia (5)	− 3.184	− 8.035	1.13E–6
			Colon adenoma (5)	− 5.101	− 10.315	1.12E–5
			Colon adenoma epithelia (5)	− 2.263	− 5.488	5.21E–5
	Skrzypczak	Colon (24)	Colorectal carcinoma (36)	− 3.771	− 8.073	4.98E–11
			Colorectal adenocarcinoma (45)	− 2.004	− 7.459	1.61E–9
	Gaedcke	Colon (65)	Rectal adenocarcinoma (65)	− 4.740	− 12.091	7.52E–20
TSPAN2	Skrzypczak 2	Colon (10)	Colon carcinoma (5)	4.984	8.441	1.86E–6
TSPAN3	Skrzypczak 2	Colon (10)	Colon carcinoma (5)	− 2.055	− 15.355	1.07E–9
			Colon adenoma (5)	− 2.550	− 10.121	8.17E–8
			Colon adenoma epithelia (5)	− 2.207	− 8.118	1.55E–5
	Hong	Colon (12)	Colorectal carcinoma (70)	− 2.156	− 13.390	1.20E–18
	TCGA	Colon (19) Rectum (3)	Rectal mucinous adenocarcinoma (6)	− 2.963	− 9.774	8.53E–9
			Cecum adenocarcinoma (22)	− 2.668	− 9.565	2.20E–12
			Rectosigmoid adenocarcinoma (3)	− 3.161	− 9.949	4.05E–5
			Rectal adenocarcinoma (60)	− 2.818	− 11.494	1.22E–14
			Colon adenocarcinoma (101)	− 2.796	− 12.471	1.02E–13
	Gaedcke	Rectum (65)	Rectal adenocarcinoma (65)	− 2.378	− 15.151	8.00E–30
	Sabates-Bellver	Colon (25) Rectum (7)	Colon adenoma (25)	− 2.488	− 7.802	2.92E–9
			Rectal adenoma (7)	− 2.036	− 3.637	0.004
Tspan5	Kaiser	Colon (5)	Cecum adenocarcinoma (17)	2.751	10.138	1.62E–9
			Rectal mucinous adenocarcinoma (4)	3.217	12.897	1.16E–5
			Colon adenocarcinoma (41)	3.123	14.960	1.71E–11
			Colon mucinous adenocarcinoma (13)	2.655	8.230	2.22E–7
			Rectosigmoid adenocarcinoma (10)	2.750	7.130	8.16E–6
			Rectal adenocarcinoma (8)	3.173	6.741	6.16E–5
	Sabates-Bellver	Colon (25) Rectum (7)	Colon adenoma (25)	2.311	9.937	1.64E–13
	Hong	Colon (12)	Colorectal carcinoma (70)	4.927	10.849	1.76E–10
	Skrzypczak	Colon (24)	Colorectal carcinoma (36)	2.176	6.260	4.60E–8
	TCGA	Colon (19) Rectum (3)	Rectal mucinous adenocarcinoma (6)	2.561	6.242	9.68E–5
			Cecum adenocarcinoma (22)	2.451	6.674	4.19E–8
			Colon adenocarcinoma (101)	2.337	9.041	1.98E–12
			Colon mucinous adenocarcinoma (22)	2.459	6.303	1.62E–7
	Gaedcke	Rectum (65)	Rectal adenocarcinoma (65)	2.335	10.930	8.15E–18
	Skrzypczak 2	Colon (10)	Colon carcinoma (5)	2.797	10.017	7.01E–7
TSPAN7	TCGA	Colon (19) Rectum (3)	Rectal adenocarcinoma (60)	− 9.405	− 23.392	9.78E–38
			Colon adenocarcinoma (101)	− 8.713	− 30.036	7.14E–41
			Colon mucinous adenocarcinoma (22)	− 12.425	− 15.720	3.82E–16
			Cecum adenocarcinoma (22)	− 8.849	− 14.091	4.39E–15
			Rectal mucinous adenocarcinoma (6)	− 10.787	− 12.008	6.84E–6
	Skrzypczak	Colon (24)	Colorectal adenocarcinoma (45)	− 4.777	− 16.217	6.86E–25
			Colorectal carcinoma (36)	− 6.249	− 12.812	1.33E–17
	Ki	Colon (28) Liver (13)	Colon adenocarcinoma (50)	− 3.124	− 10.532	3.60E–17
	Hong	Colon (12)	Colorectal carcinoma (70)	− 7.797	− 20.686	7.73E–30
	Sabates-Bellver	Colon (25) Rectum (7)	Colon adenoma (25)	− 5.074	− 15.401	4.54E–18
	Skrzypczak 2	Colon (10)	Rectal adenoma (7)	− 4.978	− 8.593	3.01E–5
			Colon carcinoma epithelia (5)	− 15.327	− 33.143	2.19E–12
			Colon adenoma epithelia (5)	− 24.778	− 29.676	4.63E–9
			Colon carcinoma (5)	− 12.648	− 37.638	7.00E–10
			Colon adenoma (5)	− 33.012	− 17.809	1.78E–5
	Notterman	Colon (18)	Colon adenocarcinoma (18)	− 3.434	− 5.711	1.18E–6
	Kaiser	Colon (5)	Rectal mucinous adenocarcinoma (4)	− 4.066	− 11.930	9.67E–6
			Colon mucinous adenocarcinoma (13)	− 4.508	− 10.063	1.05E–7
			Rectosigmoid adenocarcinoma (10)	− 4.123	− 7.975	1.18E–6
			Cecum adenocarcinoma (17)	− 3.953	− 8.093	1.53E–7
			Rectal adenocarcinoma (8)	− 3.626	− 6.516	2.57E–5
			Colon adenocarcinoma (41)	− 4.189	− 11.744	1.58E–6
	Gaedcke	Rectum (65)	Rectal adenocarcinoma (65)	− 5.640	− 20.975	3.39E–37
	Graudens	Colon (12)	Colorectal carcinoma (18)	− 2.565	− 6.685	1.57E–7
TSPAN8	Ki	Colon (28) Liver (13)	Colon adenocarcinoma (50)	− 6.065	− 8.057	8.52E–11
TSPAN9	Ki	Colon (28) Liver (13)	Colon adenocarcinoma (50)	3.175	10.975	3.82E–18
	TCGA	Colon (19) Rectum (3)	Rectosigmoid adenocarcinoma (3)	− 2.230	− 8.635	1.80E–5
TSPAN11	TCGA	Colon (19) Rectum (3)	Cecum adenocarcinoma (22)	− 3.362	− 7.883	4.28E–10
TSPAN12	Kaiser	Colon (5)	Rectal mucinous adenocarcinoma (4)	3.493	13.467	2.08E–5
			Cecum adenocarcinoma (17)	2.028	5.248	2.20E–5
TSPAN13	Gaedcke	Rectum (65)	Rectal adenocarcinoma (65)	− 2.281	− 10.970	5.47E–20
TSPAN18	Sabates-Bellver	Colon (25) Rectum (7)	Colon adenoma (25)	− 2.305	− 4.342	4.22E–5
			Rectal adenoma (7)	− 2.596	− 3.192	0.007
TSPAN22	TCGA	Colon (19) Rectum (3)	Cecum adenocarcinoma (22)	− 3.218	− 7.807	5.38E–10
			Rectosigmoid adenocarcinoma (3)	− 4.426	− 8.958	1.45E–4
			Rectal mucinous adenocarcinoma (6)	− 3.978	− 6.696	5.61E–5
	Sabates-Bellver	Colon (25) Rectum (7)	Rectal adenoma (7)	− 2.588	− 3.274	0.007
TSPAN25	Sabates-Bellver	Colon (25) Rectum (7)	Rectal adenoma (7)	− 2.037	− 6.849	9.89E–7
	TCGA	Colon (19) Rectum (3)	Rectal adenocarcinoma (60)	− 3.297	− 10.147	1.20E–15
			Colon adenocarcinoma(101)	− 2.721	− 9.230	8.52E–13
TSPAN26	Sabates-Bellver	Colon (25) Rectum (7)	Rectal adenoma (7)	− 4.014	− 6.888	5.78E–7
			Colon adenoma (25)	− 5.027	− 7.327	8.84E–10
	TCGA	Colon (19) Rectum (3)	Rectosigmoid adenocarcinoma (3)	− 3.696	− 12.747	2.29E–10
			Rectal adenocarcinoma (60)	− 3.406	− 11.758	1.27E–14
	Skrzypczak	Colon (24)	Colorectal adenocarcinoma (45)	− 2.920	− 6.590	1.46E–7
TSPAN28	Skrzypczak 2	Colon (10)	Colon carcinoma (5)	3.109	12.960	4.16E–9
			Colon adenoma epithelia (5)	2.909	8.643	3.22E–6
			Colon adenoma (5)	2.584	7.726	2.00E–5
			Colon carcinoma epithelia (5)	3.151	7.404	3.18E–6
TSPAN29	Ki	Colon (28) Liver(13)	Colon Adenocarcinoma (50)	2.054	6.211	8.79E–9
TSPAN31	TCGA	Colon (19) Rectum (3)	Cecum adenocarcinoma (22)	3.437	15.357	6.72E–19
			Colon mucinous adenocarcinoma (22)	3.249	10.684	8.81E–14
			Rectal mucinous adenocarcinoma (6)	3.348	9.458	8.89E–7
			Rectal adenocarcinoma (60)	3.181	15.893	4.15E–19
			Colon adenocarcinoma (101)	3.374	18.022	3.27E–18
			Rectosigmoid adenocarcinoma (3)	4.256	12.487	1.86E–5
	Skrzypczak 2	Colon (10)	Colon adenoma (5)	− 2.901	− 12.980	2.95E–8
			Colon carcinoma epithelia (5)	− 2.465	− 13.992	2.78E − 9
TSPAN32	TCGA	Colon (19) Rectum (3)	Rectal mucinous adenocarcinoma (6)	− 2.739	− 10.351	3.43E–8
			Cecum adenocarcinoma (22)	− 2.077	− 7.109	6.50E–9
TSPAN33	Skrzypczak 2	Colon (10)	Colon carcinoma (5)	2.104	10.676	1.37E–7

*CRC* colorectal cancer

### Expression of the TSPAN family members in gastric cancer

Differentially expressed genes between gastric cancer patients and the normal population were analyzed in the Oncomine database, and 7 data sets showed differential expression. It is interesting to note that most TSPAN proteins are highly expressed in gastric cancer. According to studies by DErrico and Cho, we found that the expression of TSPAN4, TSPAN9, TSPAN28, and TSPAN29 was higher in gastric cancer patients than it was in the normal population. The results of Chen’s research indicate that TSPAN7 is underexpressed in gastric cancer. No other TSPAN genes with differential expression were found (Table 3).

**Table 3:** Significant changes of TSPANs expression in transcription level between GC and normal gastric tissues (ONCOMINE)

Gene	Dataset	Normal (cases)	Tumor (cases)	Fold change	T-test	p-value
TSPAN4	DErrico	Gastric tissue (31)	Gastric mixed adenocarcinoma (4)	4.346	7.553	7.17E–9
		Gastric tissue (31)	Diffuse gastric adenocarcinoma (6)	3.221	4.648	1.07E–4
TSPAN7	Chen	Gastric tissue (28)	Gastric intestinal type adenocarcinoma (54)	− 2.609	− 7.307	4.42E–10
TSPAN9	Wang	Gastric tissue (15)	Gastric cancer (12)	2.679	3.592	7.19E–4
TSPAN28	DErrico	Gastric tissue (31)	Gastric mixed adenocarcinoma (4)	2.774	14.056	2.91E–7
		Gastric tissue (31)	Diffuse gastric adenocarcinoma (6)	2.059	5.574	5.30E–4
		Gastric tissue (31)	Gastric intestinal type adenocarcinoma (26)	2.052	6.976	1.49E–8
TSPAN29	Cho	Gastric tissue (19)	Gastric mixed adenocarcinoma (10)	3.537	6.371	1.82E–6
		Gastric tissue (19)	Gastric intestinal type adenocarcinoma (20)	2.369	4.277	7.00E–5
		Gastric tissue (19)	Gastric adenocarcinoma (4)	3.077	4.303	0.004
		Gastric tissue (19)	Diffuse gastric adenocarcinoma (31)	2.173	3.986	1.14E–4
	DErrico	Gastric tissue (31)	Gastric intestinal type adenocarcinoma (26)	4.263	9.674	1.14E–13
		Gastric Tissue (31)	Diffuse gastric adenocarcinoma (6)	2.321	3.924	0.001
	Wang	Gastric tissue (15)	Gastric cancer (12)	2.532	3.644	6.14E–4

*GC* gastric cancer

### TSPAN7 expression in three types of digestive system cancer cells

**Fig. 2:**
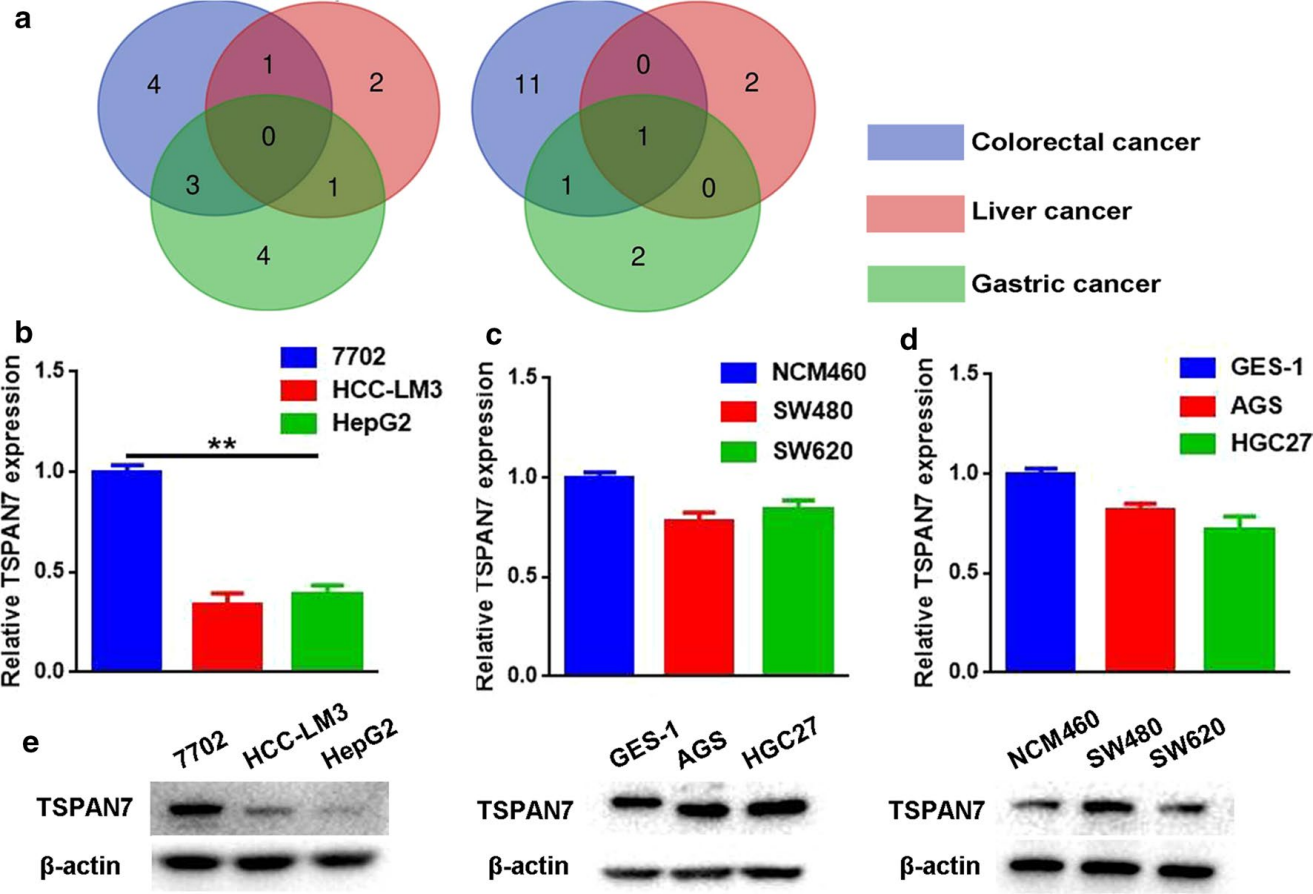
TSPAN7 expression in three types of digestive system cancer cells. **a** The Venn diagram is used to analyze members of the TSPAN family that are commonly overexpressed and underexpressed in three types of digestive system cancers. **b** qRT-PCR was employed to assess the expression of TSPAN7 in liver cancer cell lines, (**c**) colorectal cancer cell lines (**d**) and gastric cancer cell lines (n = 3). GAPDH served as an internal reference. e Western blotting was used to assess the protein levels of TSPAN7. Data represent the mean ± SD. **p < 0.01, as assessed by a Student’s t test

Bioinformatics analysis found that TSPAN7 is the only four-transmembrane protein with reduced expression in three types of digestive system cancers (Fig. 2a). However, there are no reports on the expression of TSPAN7 in liver cancer, colorectal cancer and gastric cancer. Therefore, TSPAN7 was selected for further analysis at cellular and molecular levels. We used normal human gastric epithelial cells (GES-1), normal human liver cells (7702), and normal human colon epithelial cells (NMC460) as noncancerous control lines. Using qRT-PCR and Western blotting, we evaluated the expression of TSPAN7 in digestive system cancers. We observed low levels of TSPAN7 transcripts in HCC-LM3 and HepG2 cells and confirmed these data using Western blot methods. In gastric and intestinal cancer cells, TSPAN7 expression was not significantly different from that of the normal cells (Fig. 2e).

### TSPAN7 overexpression inhibits liver cancer cell proliferation

**Fig. 3:**
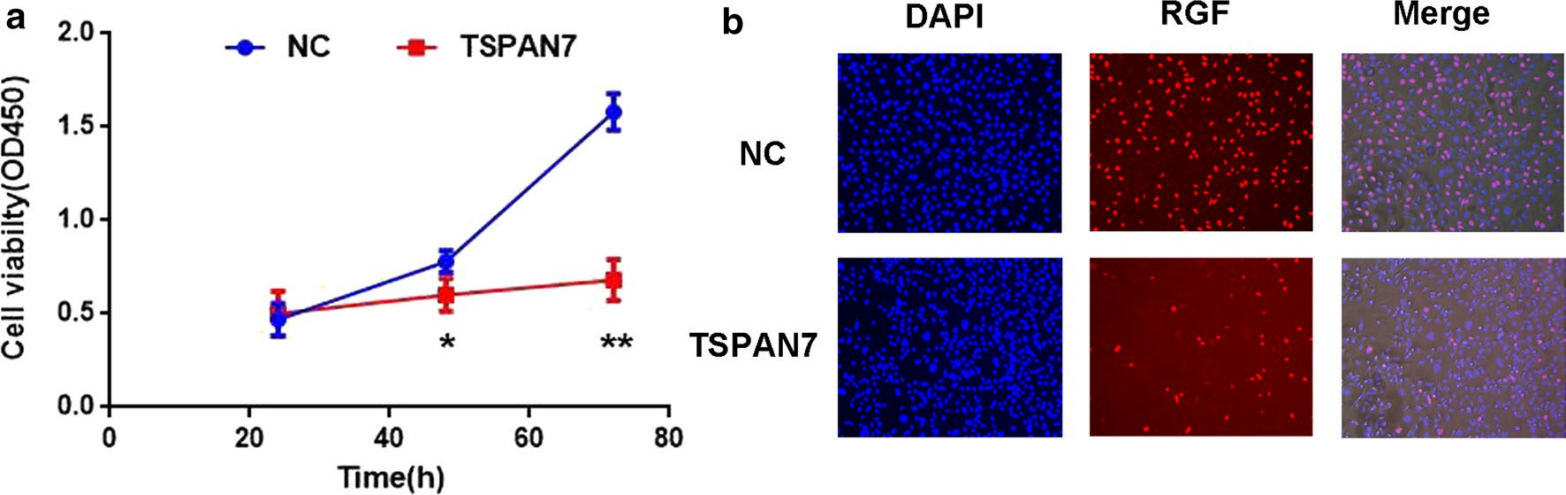
TSPAN7 overexpression inhibits liver cancer cell proliferation. **a** Tspan9 inhibits the cell viability of HCCLM3 cells, as shown by CCK-8 assays. **b** EdU assay results revealed that Tspan7 reduces the number of proliferating cells. Data represent the mean ± SD. *p < 0.05, and **p < 0.01, as assessed by a Student’s t test

We selected HCC-LM3 cells for LV-Tspan7 transfection to better understand the effect of Tspan7 on the proliferation of liver cancer cells. When using CCK-8 assays to evaluate cell viability over the course of 3 days, and we found that cell viability of the Tspan7 group was significantly lower than that of the NC group, indicating that cell viability was decreased in the treatment group (Fig. 3a). EdU experiments showed that, compared with the NC group, when TSPAN7 was overexpressed, the number of proliferating cells was reduced (Fig. 3b). These results indicate that high expression of Tspan7 significantly inhibited cell proliferation.

### TSPAN7 overexpression inhibits invasion and metastasis of liver cancer cells

**Fig. 4:**
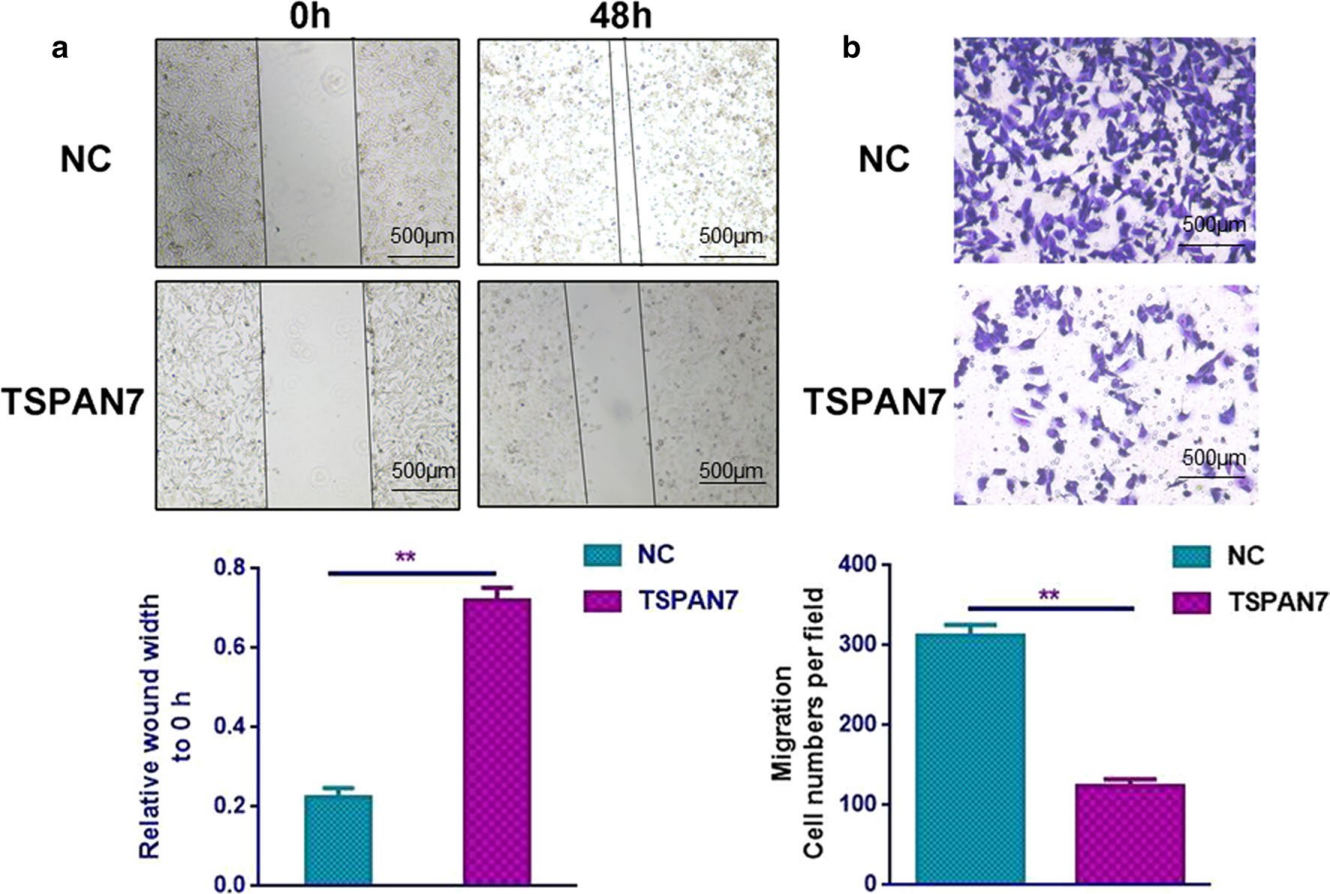
TSPAN7 overexpression inhibits invasion and metastasis of liver cancer cells. **a** In the wound-healing assay, the residual wound was much wider in the Tspan7 group than it was in the NC group after 48 h. **b** Transwell assays revealed markedly decreased cell migration and invasion ability of the Tspan7 group compared with that of the NC group. Data represent the mean ± SD. **p < 0.01, as assessed by a Student’s t test

To assess the effect of Tspan7 upregulation on cell movement, wound healing and Transwell assays were performed. We found that compared to the NC group, cells in the Tspan7 group had diminished migration into the wound, as shown by wound-healing assays (Fig. 4a). In addition, in the Transwell assay, Tspan7 overexpression significantly inhibited cell migration (Fig. 4b). These findings support the idea that Tspan7 inhibits the migration and invasion of liver cancer cells.

### Antitumor effect of TSPAN7 in tumor xenograft models

**Fig. 5:**
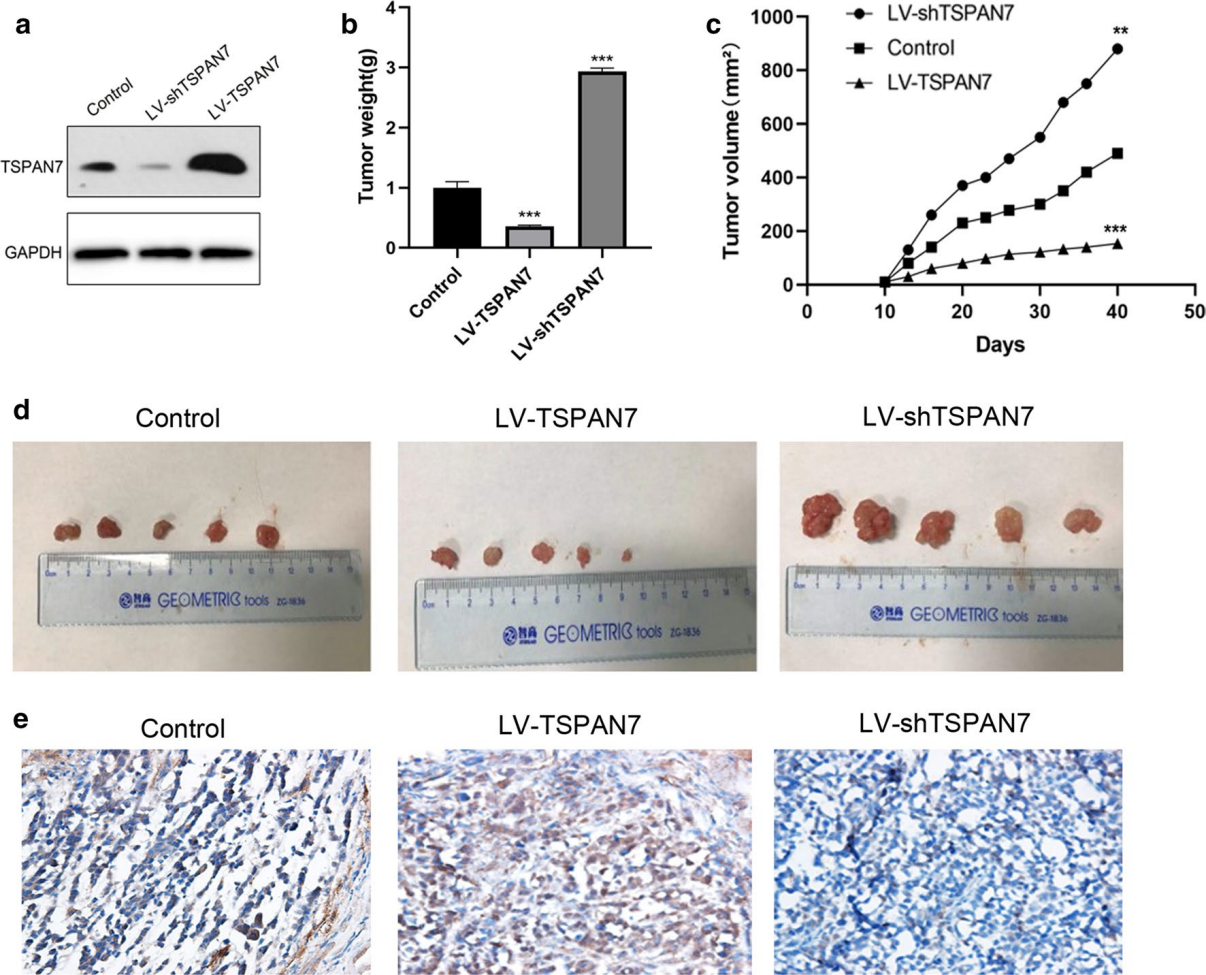
Antitumor effect of TSPAN7 in tumor xenograft models. The mice bearing HCC-LM3 xenografts were divided into three groups and treated with PBS, LV-TSPAN7, LV-shTSPAN7 once every day for a period of 40 days. **a** Western blotting was used to assess the protein expression after TSPAN7 overexpression and knockdown. **b** Tumor weight and **c** tumor volume were measured. **d** TSPAN7 protein expression in xenograft tissues. **e** HCC-LM3 cells transfected with LV-TSPAN7 and LV-shTSPAN7 were injected into nude mice (n = 5). Data represent the mean ± SD. **p < 0.01, and ***p < 0.001, as assessed by a Student’s t test

To evaluate whether TSPAN7 exerts antitumor effects in vivo, we established a nude mouse model with HCC-LM3 tumor xenografts. The results showed that overexpression of TSPAN7 had a significant effect on tumor growth inhibition, while knockdown of TSPAN7 promoted tumor growth. This result indicates the anticancer effect of TSPAN7 in vivo (Fig. 5).

## Discussion

An increasing number of experiments have been conducted around the four-transmembrane protein family and cancer; further, exploring the molecular mechanism of tumor growth and invasiveness has become the key to the development of new therapies [23–25]. However, previous studies have mostly focused on the mechanism of individual members of the TSPAN family in independent cancer types [26–29]. Therefore, a systematic analysis of the TSPAN family helps us to clarify its role in the development of tumors and guide the search for new tumor markers. To achieve this goal, we analyzed the expression of 33 TSPAN family proteins in 21 common tumors through the public database Oncomine, and we focused on their role in 3 common digestive tract tumors.

TSPAN15 is believed to enhance tumor stemness, and it has the ability to promote tumor growth and recurrence [30]. TSPAN12 can promote tumor cell proliferation and colony formation, and high expression of CD9 is considered to be related to lymph node metastasis [31, 32]. In contrast, another portion of the four-transmembrane protein family showed tumor suppression. For example, TSPAN13 has been shown to be a tumor suppressor gene in breast cancer, while TSPAN1 can inhibit the migration of alveolar epithelial cells and the EMT process [33, 34]. At the same time, other studies have found that the TSPAN proteins can regulate the phosphorylation and homodimerization of key proteins that affect the drug resistance of tumors [35]. Therefore, we believe that as a key transmembrane protein family on the cell membrane, the TSPAN protein plays a pivotal role in tumorigenesis and development. This article analyzed the expression differences of TSPAN family members in human cancer and normal tissues through an online database and explored TSPAN proteins as potential prognostic biomarkers in cancer. According to the analysis of the Oncomine public database, we found that TSPAN5 is highly expressed in liver cancer and colon cancer, TSPAN26 is highly expressed in liver cancer and gastric cancer, and TSPAN9, TSPAN28, and TSPAN29 are highly expressed in gastric and colon cancer. Interestingly, we also found that TSPAN7 was consistently underexpressed in all three digestive tract tumors. Therefore, TSPAN7 has attracted our interest for further research.

In clear cell renal cell carcinoma, the higher the TSPAN7 gene expression, the smaller the number of TSPAN7-positive blood vessels, and the less infiltration and metastasis of the cells, suggesting that TSPAN7 may act as a tumor growth inhibitor and affect tumor progression and metastasis [2]. There are reports in the literature that TSPAN 7 is upregulated in lung cancer cells, and its high expression is closely related to the poor prognosis of lung cancer patients, suggesting that TSPAN 7 plays an oncogenic role in lung cancer [36, 37]. However, the role of TSPAN7 in tumors of the digestive system has not been confirmed. We found through biochemical analysis that the expression of TSPAN7 was downregulated in gastric cancer, liver cancer and intestinal cancer, suggesting that TSPAN7 may play opposite roles in lung cancer and lung cancer. Through real-time PCR and Western blot experiments, we found that compared with normal cells, the expression of TSPAN7 in liver cancer cells was significantly reduced, while its expression in gastric and colon cancer was not significantly different from normal cells. Considering that the pathological types of gastric cancer and colorectal cancer are both adenocarcinoma, while liver cancer is cell carcinoma, the expression of TSPAN7 may be different depending on the tumor site and tissue pathology.

In subsequent studies, we mainly studied the role of Tspan7 in liver cancer cells. Since the four-transmembrane protein family also directly or indirectly participates in the process of cell proliferation, invasion and metastasis, we hope to understand what role Tspan7 plays in liver cancer cells [9, 38–40]. The results showed that the high expression of Tspan7 not only inhibited the proliferation of HCC-LM3 cells but also inhibited the invasion and metastasis of liver cancer cells. Animal experiments have also confirmed that TSPAN7 has a tumor suppressive effect in vivo. Therefore, we believe that TSPAN7 plays a tumor suppressive role in liver cancer cells. However, the downstream signaling pathways that regulate these biological functions in tumor cells and their underlying molecular mechanisms need further study.

## Conclusion

Tspan7 may be a tumor suppressor in liver cancer. As shown by our study, the high expression of Tspan7 significantly inhibited the proliferation, invasion, and metastasis of HCC-LM3 human liver cancer cells. Therefore, TSPAN7 may be a new potential biomarker that can provide a new therapeutic strategy for hepatocellular carcinoma.

## Supplementary information

**Additional file 1.** TSPAN7 lentivirus sequence.

## Data Availability

All data generated or analysed during this study are included in this published article [and its Additional files].

## Abbreviations

TSPANs: Tetraspanins
SEL: Small extracellular domain
LEL: Large extracellular loop
TEMs: Four-transmembrane protein-enriched microdomains
FBS: Fetal bovine serum
NC: Negative control
CT: Cyclic threshold method
OD: Optical density
