# Supplementary material for: Expression and function of transmembrane 4 superfamily proteins in digestive system cancers
Source: Cancer Cell Int. 2020 Jul 16;20:314. doi: 10.1186/s12935-020-01353-1 (PMC7364658; doi:10.1186/s12935-020-01353-1)
Supplement: Supplementary file 1 — Additional file 1. TSPAN7 lentivirus sequence. [file 12935_2020_1353_MOESM1_ESM.doc]

Overexpression lentiviral sequence of TSPAN7:

GTTTTTGGCTTTTTTGTTAGACGAAGCTTGGGCTGCAGGTCGACTCTAGAGGATCCCGCCACCATGGCCAGGGGCTGCCTCTGCTGCTTGAAGTACATGATGTTCCTCTTCAATTTGATATTCTGGCTCTGTGGCTGTGGGCTGCTGGGAGTGGGCATCTGGCTCTCCGTGTCCCAAGGCAACTTTGCCACCTTCTCCCCCAGCTTCCCTTCGTTGTCTGCAGCCAACCTGGTCATTGCCATAGGCACCATTGTCATGGTGACGGGCTTCCTCGGCTGCCTGGGGGCCATCAAGGAAAACAAGTGCCTCCTCCTCAGCTTTTTCATCGTCCTGTTGGTCATCCTCCTAGCAGAGCTGATCTTACTCATCCTCTTCTTTGTCTACATGGACAAGGTGAACGAGAACGCCAAGAAGGACCTGAAGGAAGGCCTGCTGCTGTACCACACCGAGAACAACGTGGGGCTGAAGAACGCCTGGAACATCATCCAGGCTGAGATGCGATGCTGTGGTGTCACTGACTACACAGACTGGTACCCAGTGCTGGGGGAGAACACGGTTCCCGACCGCTGCTGCATGGAGAACTCCCAGGGCTGCGGGCGCAACGCCACCACGCCTTTGTGGAGAACGGGCTGCTATGAAAAGGTGAAGATGTGGTTCGATGACAATAAGCACGTGCTGGGCACGGTGGGGATGTGCATCCTCATCATGCAGATCCTGGGCATGGCCTTCTCCATGACCCTCTTCCAGCACATCCACCGGACTGGTAAGAAGTACGACGCAACCGGTATGGACTACAAGGATGACGATGACAAGGATTACAAAGACGACGATGATAAGGACTATAAGGATGATGACGACAAATGAGCTAGCCTGTGGA

shRNA lentiviral sequence of TSPAN7:

TAATAATTTCTTGGGTAGTTTGCAGTTTTAAAATTATGTTTTAAAATGGACTATCATA TGCTTACCGTAACTTGAAAGTATTTCGATTTCTTGGCTTTATATATCTTGTGGAAAGGACGAAACACCGGTTCTCCGAACGTGTCACGTTTCAAGAGAACGTGACACGTTCGGAGAATTTTTGAATTCGGATCCATTAGGCGGCCGCGTGGATAACCGTATTACCGCCATGCATTAGTTATTAATAGTAATCAATTACGGGGTCATTAGTTCATAGCCCATATATGGAGTTCCGCGTTACATAACTTACGGTAAATGGCCCGCCTGGCTGACCGCCCAACGACCCCCGCCCATTGA CGTCAATAATGAC

Note: The red font is the insertion sequence, the black font is the vector sequence, and the underline is the enzyme cleavage site
